# Cross-Sectional Associations between Dietary Fat-Related Behaviors and Continuous Metabolic Syndrome Score among Young Australian Adults

**DOI:** 10.3390/nu10080972

**Published:** 2018-07-26

**Authors:** Yile Sun, Costan G. Magnussen, Terence Dwyer, Wendy H. Oddy, Alison J. Venn, Kylie J. Smith

**Affiliations:** 1 Menzies Institute for Medical Research, University of Tasmania, Hobart, TAS 7000, Australia; yile.sun@utas.edu.au (Y.S.); cmagnuss@utas.edu.au (C.G.M.); terence.dwyer@georgeinstitute.ox.ac.uk (T.D.); wendy.oddy@utas.edu.au (W.H.O.); alison.venn@utas.edu.au (A.J.V.); 2 Research Centre of Applied and Preventive Cardiovascular Medicine, University of Turku, 20520 Turku, Finland; 3 The George Institute for Global Health, University of Oxford, Oxford OX1 3QX, UK

**Keywords:** dietary fat, metabolic syndrome, cooking oil, low-fat dairy, young adults

## Abstract

Dietary guidelines recommend removing visible fat from meat, choosing low-fat options and cooking with oil instead of butter. This study examined cross-sectional associations between fat-related eating behaviors and a continuous metabolic syndrome (cMetSyn) score among young adults. During 2004–2006, 2071 participants aged 26–36 years reported how often they trimmed fat from meat, consumed low-fat dairy products and used different types of fat for cooking. A fasting blood sample was collected. Blood pressure, weight and height were measured. To create the cMetSyn score, sex-specific principal component analysis was applied to normalized risk factors of the harmonized definition of metabolic syndrome. Higher score indicates higher risk. For each behavior, differences in mean cMetSyn score were calculated using linear regression adjusted for confounders. Analyses were stratified by weight status (Body mass index (BMI) < 25 kg/m^2^ or ≥ 25 kg/m^2^). Mean cMetSyn score was positively associated with consumption of low-fat oily dressing (P_Trend_ = 0.013) among participants who were healthy weight and frequency of using canola/sunflower oil for cooking (P_Trend_ = 0.008) among participants who were overweight/obese. Trimming fat from meat, cooking with olive oil, cooking with butter, and consuming low-fat dairy products were not associated with cMetSyn score. Among young adults, following fat-related dietary recommendations tended to not be associated with metabolic risk.

## 1. Introduction

Although the cardiovascular health of adults in Australia and other developed countries has improved greatly in the last decades, cardiovascular disease (CVD) is still a leading cause of premature death and morbidity [1]. Poor diet is a modifiable behavior that can lead to risk factors for CVD, such as obesity, hypertension, hyperlipidemia, and type 2 diabetes. Dietary guidelines for the prevention of CVD recommend limiting fat intake, especially saturated fat, to reduce blood cholesterol concentration and body weight [2,3,4].

Red meat is thought to be associated with the development of CVD due to the high proportion of saturated fat. However, after trimming visible fat, red meat such as beef and lamb contains up to 5% total fat [5], which is no more than that of some white meats [6]. Studies examining the association between red meat consumption and CVD risk have reported inconsistent results. The Nurses’ Health Study reported that red meat consumption was positively associated with an increased risk of nonfatal myocardial infarction or fatal coronary heart disease (CHD) [7] whereas a meta-analysis in 2010 found no association between consumption of red meat and incidence of CHD or diabetes risk [8].

Dairy products are also believed to increase CVD risk as the fat in dairy products is mainly saturated. However, different saturated fats have different effects on plasma lipids and lipoproteins and some may protect against CVD risk factors [9]. The food matrix may also modify the effect of dairy fat on CVD outcomes [10,11]. A recent review, funded by the dairy industry, found that most studies reported either an inverse association or no association between consumption of dairy products and CVD risk [12]. Studies comparing the effect of full fat and reduced fat dairy on CVD risk are limited [12].

Oils derived from plants have been promoted as being healthier than animal fat, with recommendations to replace animal fats with oil when cooking [13]. The composition of monounsaturated and polyunsaturated fatty acids varies among different vegetable oils. Replacing saturated fatty acids with polyunsaturated fatty acids, found in plant-based oils, can result in an increased risk of death from CVD if the polyunsaturated fats are not omega-3s [14]. This suggests that substitution of animal fat with vegetable oils may not necessarily reduce CVD risk.

Most studies have examined whether following advice for fat-related dietary behaviors is associated with lower risk of CVD among middle-aged to elderly individuals. It is important to determine whether the same associations exist among young adults. This study examined whether young adults who usually trimmed fat from meat, consumed low-fat products, and used oil for cooking had lower CVD risk than those who did not follow these practices. CVD risk was assessed using a continuous metabolic syndrome (cMetSyn) score.

## 2. Materials and Methods 

### 2.1. Study Population

The Childhood Determinants of Adult Health (CDAH) Study is a follow-up of the 1985 Australian Schools Health and Fitness Survey, which included 8498 children aged 7–15 years [15]. After tracing participants during 2001–2002, 5170 were enrolled in the CDAH study. During 2004–2006, 2410 participants aged 26–36 years attended one of 34 clinics around Australia. This study was conducted according to the guidelines laid down in the Declaration of Helsinki and all procedures involving human participants were approved by the Southern Tasmania Health and Medical Ethics Committee (H6020). Written informed consent was obtained from all participants.

### 2.2. Dietary Assessment

Diet was assessed using a food frequency questionnaire and a food habits questionnaire [16]. Whether visible fat was removed from meat before eating was assessed using the question ‘how often is the meat you eat trimmed of fat either before or after cooking’, with response options: ‘never/rarely’, ‘sometimes’, ‘usually’, and ‘I don’t eat meat’. How often participants used specific types of fat for cooking was assessed using the question ‘when cooking, how often do you or the person who cooks your food use the following (olive oil, canola or sunflower oil, butter)?’ Response options were ‘never/rarely’, ‘sometimes’, ‘usually’, and ‘don’t know’. Consumption of low or reduced fat dairy products (cream, ice-cream, cheddar-type cheese), oily salad dressing and spreads was assessed using the question ‘when you eat the following products, how often do you eat a low/reduced fat variety?’ Response options were ‘never/rarely’, ‘sometimes’, ‘usually’, and ‘I don’t eat this food’.

### 2.3. Anthropometric Measurements

All anthropometric measurements were made by trained staff using standardized techniques. Weight was measured without heavy clothing or shoes using a Heine portable scale (Heine, Dover, NH, USA) and recorded to the nearest 0.1 kg. Height was measured without shoes using a portable Leicester stadiometer (Invicta, Leicester, UK) and recorded to the nearest 0.5 cm. Waist circumference was measured using a non-stretch tape at the narrowest point between the lower costal border and the iliac crest. Participants were standing, and the measurement was taken at the end of gentle expiration. Waist circumference was measured three times, the mean of the three measurements was used. Body mass index (BMI, kg/m^2^) was calculated as weight (kg)/height (m^2^).

### 2.4. Blood Chemistry

Venous blood samples were taken from the antecubital vein of participants after an overnight fast. Triglycerides, total cholesterol, high density lipoprotein (HDL) cholesterol and blood glucose were determined by enzymatic procedures using an Olympus AU5400 automated analyzer (Olympus Optical, Tokyo, Japan) [17].

### 2.5. Blood Pressure

Blood pressure was measured from the right brachial artery three times with a 1-min interval between each measurement, using a digital automatic monitor (Omron HEM907, Omron Healthcare Inc., Kyoto, Japan). The mean of the three measurements was used. Mean arterial pressure was calculated as 1/3((2 × diastolic) + systolic).

### 2.6. Continuous Metabolic Syndrome (cMetSyn) Score

A cMetSyn score was created using the methods described by Wijndaele et al. [18,19]. A continuous score was used because cardiometabolic risk increases as the number of risk factors increases and it prevents the need to dichotomize continuous outcomes. All risk factors in the harmonized definition of the metabolic syndrome [20] were normalized and sex-specific principal component analysis with varimax rotation was applied. Two principal components with eigenvalue >1.0 were identified (fasting glucose and mean arterial pressure; waist circumference, triglycerides and HDL cholesterol). These two principal components explained 26% and 34% of the variance in men and 25% and 31% of the variance in women [21]. The cMetSyn score was calculated by summing the principal component scores and weighting them based on the relative proportion of variance explained. A higher score indicates higher risk. The score ranged from −2.6 to 3.1. To determine if the continuous score was appropriate at identifying those with higher metabolic risk, cMetSyn scores were compared between those with and without the harmonized definition of the metabolic syndrome. The mean (SD) cMetSyn scores were higher among participants classified as having metabolic syndrome than those without metabolic syndrome for both men (metabolic syndrome 1.19 (0.38), no metabolic syndrome −0.14 (0.60)), and women (metabolic syndrome 1.68 (0.55), no metabolic syndrome 0.06 (−0.63)).

### 2.7. Covariates

Participants reported their age, occupation, highest level of education, smoking status, and marital status. The food frequency questionnaire was used to estimate usual consumption of 127 food and beverages over the previous 12 months. For each item, participants chose one of nine response options ranging from ‘never or <1/month’ to ‘6+ times/day’. Daily equivalents were calculated for each item, assuming one serve was consumed at each eating occasion [16]. Usual consumption of hot takeaway food was reported [16]. Usual daily intake of fruit and vegetables were estimated using short questions [16,22]. The number of standard alcoholic drinks (10 g of alcohol) consumed per week was estimated from the frequency of nine alcohol beverages and their average alcohol concentration [16]. Diet quality was estimated using a Dietary Guideline Index, which assessed compliance with the Australian Dietary Guidelines [3]. The Dietary Guideline Index included 15 components, scored 0–10, with a higher score indicating greater compliance [23,24]. Scores for each component were summed to give the total Dietary Guideline Index score (potential range: 0–150) [25]. Participants reported if they were currently following a weight loss diet or a fat-modified diet. The long version of the International Physical Activity Questionnaire [26] was used to estimate leisure-time physical activity (minutes/week). As an objective measure of physical activity, pedometers (Yamax Digiwalker SW200) were worn for seven days to estimate daily steps.

### 2.8. Statistical Methods

Participant characteristics are presented as mean (SD) or median (25th, 75th percentiles) for continuous variables and *n* (%) for categorical variables. Associations between dietary behaviors and mean cMetSyn score were calculated using linear regression. The dependent variable was transformed where necessary to improve normality of residuals. To determine if the association varied by weight status (healthy weight BMI < 25 kg/m^2^ versus overweight or obese BMI ≥ 25/m^2^) an interaction term was added to each model and stratified by weight status when an interaction was found (*p* < 0.05). Men and women were analyzed together and sex interactions were examined. The results were stratified by sex when a sex interaction was found (*p* < 0.05). Model 1 adjusted for age and sex (if no sex interaction). Fat-related behaviors that were found to be associated (*p* < 0.1) with cMetSyn score in model 1, were adjusted for other covariates in model 2. Covariates were included in model 2 if they changed the coefficient of the predictor by at least 10%. The covariates considered were: marital status, education, occupation, smoking status, daily fruit and vegetable consumption, weekly alcohol consumption, leisure-time physical activity, and daily steps. When low-fat products were the predictor of interest, model 2 was also adjusted for intake (daily equivalent) of that item (e.g., consumption of low-fat cheese was adjusted for cheese intake). When the predictor was trimming fat from meat, model 2 was adjusted for intake of meats considered to be high in fat if the fat had not been removed [16]. When type of oil used for cooking was the predictor, model 2 was adjusted for takeaway food consumption as takeaway foods can contain large amounts of oil and the participant may not know what type of oil was used. In a sensitivity analysis, the Dietary Guideline Index was added to model 2 to determine if the association was mediated by diet quality. Additional sensitivity analyses adjusted for being on a weight loss or fat-modified diet. Participants were excluded from the analysis examining type of fat used for cooking if they did not know how often that fat was used (olive oil *n* = 11, canola oil *n* = 32, butter *n* = 8). All statistical analyses were conducted with STATA software (version 13.1, 2016, StataCorp., College Station, TX, USA).

## 3. Results

In total, 2084 participants answered the food habits questionnaire and had data required to calculate the cMetSyn score. Participants were excluded if they were pregnant (*n* = 7) or used lipid lowing medication (*n* = 6), leaving 2071 participants for the analysis.

The characteristics of the participants are presented in Table 1. Compared with the Australian population of 25–34-year-olds, the CDAH sample had a higher percentage of participants who were married or living as married (57% men, 64% women in the general population [27]) and employed as professionals or managers (40% men, 38% women in the general population [28]). The percentage of participants who were overweight or obese (BMI ≥ 25 kg/m^2^) was comparable to the Australian population of similar age (58% men, 35% women [29]). Mean (SD) cMetSyn score was −0.01 (−0.70) for men and −0.01 (−0.71) for women.

The analysis was stratified by weight status due to significant interactions between weight status and all fat-related behaviors. Most participants reported usually trimming fat from meat before or after cooking (Table 2, 63% healthy weight, 78% overweight/obese). Participants who were overweight or obese tended to consume low-fat products more frequently than those who were healthy weight. Most participants usually used olive oil for cooking (62% healthy weight, 60% overweight/obese) whereas the proportion of participants who usually used canola oil (10% healthy weight, 10% overweight or obese) or butter (8% healthy weight, 7% overweight or obese) was low. Compared to healthy weight participants (3.3%), a higher percentage of those who were overweight or obese (8.2%) reported they were on a weight loss diet, whereas the percentage on a fat-modified diet was similar (11.9% healthy weight, 10.6% overweight/obese).

Among participants who were healthy weight, frequency of using low-fat oily dressing was the only fat-related behavior that was significantly associated with cMetSyn (Table 3). Participants who never or rarely used low-fat oily dressing had lower cMetSyn scores than those who usually used it. The association remained significant after adjusting for education, daily fruit and vegetable consumption, weekly alcohol consumption, smoking status and intake of oil and creamy dressings. Further adjustment for diet quality, being on a weight loss or fat-modified diet did not attenuate the results.

Among participants who were overweight or obese, participants who usually used canola/sunflower oil for cooking had higher cMetSyn scores than those who never/rarely used it (Table 3). The association remained after adjusting for smoking status and takeaway food consumption, and after adjusting for diet quality, being on a weight loss or fat-modified diet in the sensitivity analyses. A significant sex interaction was found for cooking with butter among those who were overweight or obese, with a trend for a higher score among males who more frequently used butter for cooking. Participants who were overweight or obese and usually consumed whole milk had higher cMetSyn scores than those who usually consumed skim or low/reduced fat milk. However, the association was attenuated and no longer statistically significant after adjusting for education and occupation. 

## 4. Discussion

This study examined whether fat-related eating behaviors (trimming fat from meat, consuming low-fat dairy, type of fat used for cooking) were associated with a cMetSyn score among young Australian adults. Healthy fat-related dietary behaviors were reported by a higher percentage of participants who were overweight or obese than those who were healthy weight. This may be a result of reducing fat intake to try to lose weight or greater pressure to report socially desirable answers. The cMetSyn score was higher among healthy weight participants who reported more frequent use of low-fat oily dressings and among participants who were overweight or obese and used canola oil more frequently for cooking. The other fat-related dietary behaviors were not associated with cMetSyn.

Trimming fat from meat was not associated with the cMetSyn score. This findings is consistent with previous studies that have reported no significant relationship between intake of animal fat [30,31,32] or saturated fat [33] and the risk of CHD. In a meta-analysis of 17 cohort studies and three case-control studies, red meat intake was not associated with CHD or Type 2 diabetes [8]. It is worth noting in that study the effects of lean and non-lean cuts of meat were not examined separately. Other studies that investigated lean red meat also found no negative effects on lipid levels, thrombotic risk factors, markers of oxidative stress or blood pressure [34]. In contrast, a recent review of systematic reviews and meta-analyses recommended reducing meat-derived saturated fat for CVD health [4].

Among healthy weight participants, more frequent use of low-fat oily dressing was associated with a higher cMetSyn score. Oily dressings are usually high in vegetable oils, which are recommended for cardiometabolic health. Although low-fat dressings usually have a lower energy content, the healthy oils have been replaced with sugar, starch and salt, components which are detrimental to cardiometabolic health [35].

Our study found using olive oil for cooking was not associated with cMetSyn score. These results contrast those from studies of older participants that found higher olive oil to associate with better cardiometabolic health. In the PREvención con DIeta MEDiterránea (PREDIMED) Study (*N* = 7216, 55–80 years old), the risk of developing CVD during the 4.8 year follow-up was 35% lower among participants with the highest baseline olive oil consumption, compared to those in the lower third [36]. In a Spanish cohort of the European Prospective Investigation into Cancer and Nutrition (EPIC) study (*N* = 40,142, 29–69 years old) [37], olive oil, especially extra virgin olive oil, was related to a reduced risk of CHD events during a mean follow-up of 10.4 years. A meta-analysis of nine studies found an inverse relationship between olive oil consumption and stroke (and with stroke and CHD combined), but no association was found for CHD [38]. In a cross-sectional study of Spanish adults (*N* = 4572, 18 years and older), olive oil consumption was associated with a lower risk of obesity, impaired glucose regulation and hypertriglyceridemia than consumption of sunflower oil [39]. The younger age of our study participants and assessed use of olive oil for cooking, not overall intake, could explain the observed differences with previous findings.

Among participants who were overweight or obese, higher consumption of canola/sunflower oil was associated with higher mean cMetSyn score. These findings are in contrast with a review, funded by the Canola industry [40], in which most studies found canola oil was associated with beneficial effects on biomarkers of CVD risk (total cholesterol, LDL-cholesterol, and inflammatory biomarkers) compared with saturated fat. Sunflower oil appeared to have similar effects on plasma lipids as canola oil whereas olive oil was less protective [40]. One potential reason for the conflicting findings is that most of the studies included in that review used raw oil which restricts the interpretation of these studies, because canola oil is mostly used for frying, and heat can cause the loss of some of the beneficial components such as α-linolenic acid [41].

We found that using butter for cooking was not significantly associated with cMetSyn score. However, the number of participants who usually used butter for cooking was low and there was a trend for more frequent use of butter to be associated with a higher cMetSyn score among males who were overweight or obese. Previous studies have reported that butter consumption was not associated with Ischemic Heart Disease events [42], incident CVD [43] or stroke [44].

Consumption of low-fat dairy products was not associated with cMetSyn scores after adjusting for potential confounders, which is consistent with recent studies [45,46,47]. In a multicenter cross-sectional study of 4827 French adults aged over 35 years, no association was found between high-fat dairy intake and lipid profile or risk of mortality [45]. In a Netherlands cohort (*N* = 120,852, 55–69 years old), intake of full-fat or low-fat milk products did not predict the risk of Ischemic Heart Disease or stroke in men or women during the 10-year follow-up [46]. A meta-analysis assessing six prospective cohort studies [47] also indicated no association between total high-fat and low-fat dairy consumption and the risk of CHD. Among 2907 adults aged 65 years and older from the USA, serial measures of biomarkers for dairy fat were not significantly associated with incident CVD after 22 years of follow-up [48]. Higher circulating heptadecanoic acid was associated with lower risk of CVD and stroke mortality [48]. In the Nurses’ Health Study [49] (80,082 women, aged 34–59 years), no association was found between high-fat or low-fat dairy consumption and risk of major CHD events during the 14-year follow-up; however, a higher ratio of high-fat to low-fat dairy consumption was associated with greater CHD risk. Whole-fat milk was also found to be positively associated with CHD risk in that study. 

This study had limitations. Self-reported dietary data may be subject to error due to recall or reporting of socially desirable answers. The present study made no distinctions between canola and sunflower oil, or different varieties of olive oil. Data on participants use of oil was only collected in relation to cooking, not other uses such as dressings. The cross-sectional design means the direction of the association cannot be determined. It is possible that participants who are concerned about their metabolic health (e.g., have been informed they have high cholesterol) or have a family history of CVD may have recently changed their behavior to improve their cardiometabolic health [50]. In addition, the study focused on components of the harmonized definition of the metabolic syndrome [20] and did not include other risk factors such as non-alcoholic fatty liver disease, which is strongly associated with cardiometabolic health [51]. Finally, data was only collected on the frequency of the eating behaviors rather than the amount consumed, therefore the overall fat composition of the diets, such as total, saturated and unsaturated fat intake is unknown. However, estimating portion sizes is difficult and prone to measurement error and the major variance of food intake is explained by frequency alone [52].

Strengths of the study include studying a relatively healthy population, which avoids possible treatment and disease effects on the outcomes of interest. Most existing studies have focused on middle-aged and elderly populations whereas this study examined associations between fat-related eating behaviors and CVD risk among young adults. In addition, the CDAH study is a multicenter study with a large sample size from across Australia, with generalizability to healthy young Australian adults. The analyses were adjusted for various lifestyle and dietary factors.

## 5. Conclusions

In this cross-sectional study of young Australian adults, a higher cMetSyn score was associated with more frequent consumption of low-fat oily dressings among participants who were healthy weight and frequent use of canola oil for cooking among those who were overweight or obese. Trimming fat from meat, cooking with olive oil and consumption of low-fat dairy products was not associated with metabolic risk.

## Figures and Tables

**Table 1:** Sociodemographic, lifestyle and cardiometabolic characteristics of participants from the Childhood Determinants of Adult Health study, aged 26–36 years old.

Characteristic	Men (*N* = 1021) ^a^		Women (*N* = 1050) ^a^	
	*n*	%	*n*	%
Education				
University	391	38.5	507	48.3
Vocational	367	36.1	258	24.6
School only	259	25.5	284	27.1
Marital status				
Single	330	32.3	334	31.8
Married/living as married	691	67.7	716	68.2
Occupation				
Profession/Manager	586	58.4	539	52.0
White collar	78	7.8	281	27.1
Blue collar	310	30.9	52	5.0
Not in labor force	30	3.0	165	15.9
Smoking status				
Never	584	57.4	584	55.8
Former	183	18.0	242	23.1
Current	251	24.7	221	21.1
Weight status				
Healthy weight (<25 kg/m^2^)	392	38.4	662	63.1
Overweight (25 kg/m^2^–<30 kg/m^2^)	470	46.0	245	23.3
Obese (≥30 kg/m^2^)	159	15.6	143	13.6
	Mean	SD	Mean	SD
Age, years	31.6	2.6	31.4	2.6
Average daily steps	9221	3532	8978	3041
Leisure-time physical activity (minutes/week)	120	0–250	120	20–230
Cardiometabolic risk factors				
Waist circumference (cm)	89.3	10.3	77.9	11.1
Fasting glucose (mmol/L)	5.2	0.4	4.8	0.4
HDL cholesterol (mmol/L)	1.3	0.3	1.5	0.3
Systolic blood pressure (mmHg)	125	11	111	10
Diastolic blood pressure (mmHg)	75	9	70	9
cMetSyn ^b^	−0.01	−0.70	−0.01	−0.71
	Median	IQR ^c^	Median	IQR ^c^
Triglycerides (mmol/L)	1.0	0.7–1.6	0.8	0.6–1.1

^a^ Numbers do not always equal the total due to missing data. ^b^ Continuous metabolic syndrome score, a higher score indicates higher cardiometabolic risk. ^c^ IQR, interquartile range (25th, 75th percentiles).

**Table 2:** Distribution of fat-related eating behaviors, stratified by weight status, from the Childhood Determinants of Adult Health study.

Fat-Related Eating Behavior	BMI < 25 kg/m^2^ (*N* = 1054) ^a^		BMI ≥ 25 kg/m^2^ (*N* = 1017) ^a^	
	*n*	%	*n*	%
Trim fat from meat				
Don’t eat this food	56	5.4	18	1.8
Usually	746	71.5	699	69.5
Sometimes	149	14.3	194	19.3
Rarely/never	92	8.8	95	9.4
Low-fat cream				
Don’t eat this food	177	16.8	158	15.6
Usually	276	26.2	306	30.2
Sometimes	174	16.5	208	20.5
Rarely/never	425	40.4	341	33.7
Low-fat ice-cream				
Don’t eat this food	85	8.1	71	7.0
Usually	215	20.5	237	23.4
Sometimes	214	20.4	246	24.2
Rarely/never	536	51.1	461	45.4
Low-fat cheddar-type cheese				
Don’t eat this food	31	3.0	33	3.3
Usually	305	29.1	358	35.3
Sometimes	249	23.7	269	26.5
Rarely/never	465	44.3	355	35.0
Low-fat oily dressing				
Don’t eat this food	188	18.3	194	19.1
Usually	322	31.4	358	35.3
Sometimes	207	20.2	194	19.1
Rarely/never	308	30.1	269	26.5
Low-fat spread				
Don’t eat this food	47	4.5	35	3.5
Usually	401	38.1	456	45.0
Sometimes	236	22.4	242	23.9
Rarely/never	368	35.0	280	27.6
Olive oil for cooking				
Usually	645	62.0	551	54.7
Sometimes	283	27.2	324	32.2
Rarely/never	113	10.9	132	13.1
Canola/sunflower oil for cooking				
Usually	99	9.7	102	10.4
Sometimes	325	31.8	312	31.7
Rarely/never	597	58.5	569	57.9
Butter for cooking				
Usually	77	7.5	67	6.8
Sometimes	482	46.6	432	43.6
Rarely/never	475	45.9	491	49.6
Milk usually consumed				
Skim milk	134	12.9	153	15.7
Low/reduced fat	512	49.4	449	46.0
Whole milk	391	37.7	375	38.4

^a^ Totals may not add to 100% due to rounding.

**Table 3:** Adjusted mean (SE) continuous metabolic syndrome score by fat-related eating behaviors, stratified by weight status, for participants from the Childhood Determinants of Adult Health study.

Fat-Related Eating Behavior	I Don’t Eat This Food			Usually			Sometimes			Never/Rarely			*p*-Trend	
	*n*	Mean ^a^	(SE)	*n*	Mean ^a^	(SE)	*n*	Mean ^a^	(SE)	*n*	Mean ^a^	(SE)	Model 1 ^b^	Model 2
BMI < 25 kg/m^2^														
Trim fat from meat	56	−0.54	(0.07)	746	−0.46	(0.03)	149	−0.42	(0.05)	92	−0.50	(0.06)	0.690	---- ^c^
Low-fat cream	177	−0.48	(0.04)	276	−0.44	(0.04)	174	−0.48	(0.04)	425	−0.45	(0.03)	0.771	---- ^c^
Low-fat ice-cream	85	−0.47	(0.06)	215	−0.44	(0.04)	214	−0.41	(0.04)	536	−0.49	(0.03)	0.353	---- ^c^
Low-fat cheese	31	−0.51	(0.09)	305	−0.43	(0.04)	249	−0.40	(0.04)	465	−0.50	(0.03)	0.184	---- ^c^
Low-fat oily dressing	188	−0.43	(0.04)	322	−0.42	(0.04)	207	−0.47	(0.04)	308	−0.53	(0.03)	0.010	0.013 ^d^
Low-fat spread														
-Males	17	−0.58	(0.12)	122	−0.41	(0.05)	104	−0.41	(0.05)	149	−0.54	(0.04)	0.125	---- ^c^
-Females	30	−0.49	(0.10)	279	−0.28	(0.03)	132	−0.29	(0.05)	219	−0.33	(0.04)	0.836	---- ^c^
Olive oil for cooking	--- ^e^	--- ^e^	--- ^e^	645	−0.48	(0.03)	283	−0.42	(0.04)	113	−0.45	(0.05)	0.209	---- ^c^
Canola/sunflower oil for cooking	--- ^e^	--- ^e^	--- ^e^	99	−0.44	(0.06)	325	−0.44	(0.03)	597	−0.49	(0.03)	0.223	---- ^c^
Butter for cooking	--- ^e^	--- ^e^	--- ^e^	77	−0.49	(0.06)	482	−0.48	(0.03)	475	−0.45	(0.03)	0.401	---- ^c^
				Skim milk			Low/reduced fat milk			Whole milk				
Milk usually consumed	--- ^e^	--- ^e^	--- ^e^	134	−0.46	(0.05)	512	−0.46	(0.03)	391	−0.46	(0.03)	0.929	---- ^c^
BMI ≥ 25 kg/m^2^														
Trim fat from meat	18	0.003	(0.15)	699	0.25	(0.03)	194	0.28	(0.05)	95	0.26	(0.07)	0.394	---- ^c^
Low-fat cream	158	0.20	(0.05)	306	0.26	(0.04)	208	0.23	(0.05)	341	0.28	(0.04)	0.356	---- ^c^
Low-fat ice-cream	71	0.22	(0.08)	237	0.26	(0.05)	246	0.27	(0.04)	461	0.25	(0.03)	0.944	---- ^c^
Low-fat cheese	33	0.38	(0.12)	358	0.25	(0.04)	269	0.29	(0.04)	355	0.22	(0.04)	0.384	---- ^c^
Low-fat oily dressing	194	0.26	(0.05)	358	0.22	(0.04)	194	0.24	(0.05)	269	0.29	(0.04)	0.489	---- ^c^
Low-fat spread	35	0.13	(0.11)	456	0.24	(0.04)	242	0.29	(0.04)	280	0.25	(0.04)	0.416	---- ^c^
Olive oil for cooking	--- ^e^	--- ^e^	--- ^e^	551	0.22	(0.03)	324	0.30	(0.04)	132	0.29	(0.06)	0.101	---- ^c^
Canola/sunflower oil for cooking	--- ^e^	--- ^e^	--- ^e^	102	0.35	(0.07)	312	0.30	(0.04)	569	0.20	(0.03)	0.007	0.008 ^f^
Butter for cooking														
-Males	--- ^e^	--- ^e^	--- ^e^	49	0.38	(0.09)	272	0.28	(0.04)	287	0.21	(0.04)	0.060	0.077 ^g^
-Females	--- ^e^	--- ^e^	--- ^e^	18	0.29	(0.15)	160	0.57	(0.05)	204	0.48	(0.05)	0.792	---- ^c^
				Skim milk			Low/reduced fat milk			Whole milk				
Milk usually consumed	--- ^e^	--- ^e^	--- ^e^	153	0.17	(0.06)	449	0.22	(0.04)	375	0.29	(0.04)	0.042	0.295 ^h^

^a^ Mean and SE are reported for model 1. Higher score indicates higher cardiometabolic risk. ^b^ Model 1 adjusted for age and sex, except when there was a sex interaction, then the analysis was stratified by sex. ^c^ no additional adjustments were made for model 2 as *p*-value for model 1 was not <0.1. Model 2 made additional adjustments for ^d^ education status, daily fruit and vegetable consumption, ^e^ the response option “I don’t eat this food” was not available for this variable; weekly alcohol consumption, smoking status, intake of oily and creamy dressings; ^f^ smoking status, takeaway food consumption; ^g^ occupation; ^h^ education, occupation.
